# A Study on Distortion Estimation Based on Image Gradients

**DOI:** 10.3390/s22020639

**Published:** 2022-01-14

**Authors:** Sin Chee Chin, Chee-Onn Chow, Jeevan Kanesan, Joon Huang Chuah

**Affiliations:** Department of Electrical Engineering, Faculty of Engineering, Universiti Malaya, Kuala Lumpur 50603, Malaysia; imsinchee@gmail.com (S.C.C.); jievan@um.edu.my (J.K.); jhchuah@um.edu.my (J.H.C.)

**Keywords:** noise, distortion estimation, multiple corruption estimation, image gradients

## Abstract

Image noise is a variation of uneven pixel values that occurs randomly. A good estimation of image noise parameters is crucial in image noise modeling, image denoising, and image quality assessment. To the best of our knowledge, there is no single estimator that can predict all noise parameters for multiple noise types. The first contribution of our research was to design a noise data feature extractor that can effectively extract noise information from the image pair. The second contribution of our work leveraged other noise parameter estimation algorithms that can only predict one type of noise. Our proposed method, DE-G, can estimate additive noise, multiplicative noise, and impulsive noise from single-source images accurately. We also show the capability of the proposed method in estimating multiple corruptions.

## 1. Introduction

Image noise is one of the major sources of corruption of an image. Severe noise in an image can cause information loss. Image noises are described as the incorrect bit information on the image due to several reasons, for example a false bit-flip is caused by the thermal noise in the internal circuitry of the camera, and random noises are due to the random exposure of the light sensor in the camera to radiation when taking a photo. The nature and strength of noise have a high correlation with the surrounding environment. A good noise type and strength estimator is needed to study the relationship between the surrounding environment and the resultant image we received. For example, X-ray projection suffers from severe noise corruption and noise distribution, commonly modeled as Poisson noise or Rician noise. The standard deviation of noise has a high correlation with the X-ray voltage and current because reducing the power of the X-ray will cause the Bremsstrahlung curve to change [1].

Throughout the years, several noise models have been proposed to model the noise observed in a noisy image. Additive White Gaussian Noise (AWGN) is commonly used as the random noise in both one-dimensional signal and image noise. Salt-and-pepper noise is proposed as the false bit-flip effect shown in images when there is thermal noise present. Noise modeling is extremely important as the noise model’s parameter can be used as a reference for the nature of the noise observed in the image. Image Quality Assessment (IQA) is a systematic way of grading the image noise corruption level by comparing the score predicted by the algorithm and the Mean Opinion Score (MOS) of several subjects ranging from experts to novices. IQA is designed to accommodate all types of distortions and give an overall score. It is hard to judge the noise strength and severity of the corruption of the image from the predicted score of IQA because there might be more than one type of distortion present in the noisy image and the correlation between the IQA score across different types of noise is different. As image noise is randomly distributed with a specific distribution, the IQA score is often non-reproducible. A noise model parameter estimation is a good representation of the nature of noise. Hence, an inverse process is required to estimate the noise parameters.

A picture is worth a thousand words. An image can describe the environment perfectly without the need for description and explanation. Feature extraction is a technique to extract information from the input signal. Feature extraction can downsample the input signal by extracting information that we need for further processing. Recent developments in machine learning have explored different architectures for performing deep feature extraction in tasks such as classification, object detection, and anomaly detection [2,3,4,5]. With the emergence of 5G technology, the boom in the volume of data has made the deep learning approach feasible because machine learning requires an abundance of data to perform the fine-tuning of the millions of parameters in the neural network. Developments in image recovery such as U-net and Hi-Net have taken a supervised approach in performing image manipulation, such as denoising, deblurring, etc. [6,7]. The architecture of these autoencoder networks downsample the vector space and upsample it again to produce images that are free of noise. Recently, the extension of U-net’s capability to performing denoising in medical images has been studied. To train such a network, the ground truth–noisy pair must be known. Unfortunately, such an image dataset is very hard to achieve due to reasons such as privacy issues and health issues. Besides from medical images, other types of ground truth–noisy image pairs that are hard to collect are motion blur and glare. Deblur GAN, which is designed to perform motion deblurring of motion blur images through an adversarial training approach, requires an image pair to conduct training [8,9]. Motion blur images are hard to obtain because the experiments are hard to replicate, especially on natural images.

A recent study showed the possibility of using synthetic data to generate the ground truth–noisy image pair by using a simulator that closely resembles the actual environment [10]. Reference [11] obtained the image pair of glare images and performed glare removal. The result showed significant improvement because more data were used for training. Data acquisition in a simulated environment is less costly and versatile compared to real-life data collection. A well-designed simulator allows the user to change the resolution, physical limits, and noise of the acquired data. The data obtained are no longer restricted to the physical limitations such as being unable to place two cameras at the same spot while taking a photo at different shutter speeds (data acquisition of the motion blur image) and a virtual environment allowing data collection all day long. The process of data collection does not require human supervision in a simulated environment. However, the data obtained from a virtual environment cannot be used as the training dataset for any machine learning application because noise is absent from the acquired dataset. The correct noise injection is applied to the synthetic data obtained through the simulator. Augmented data with noise injection need to be similar to the actual noise seen in real-life training data.

Besides creating realistic synthetic data and studying the environment, noise strength estimation is also important in noise suppression. Image denoising is a heavily studied field in quality image acquisition. The goal of image denoising is to preserve the spatial domain and to suppress the range domain. Some prior information of the range domain is needed such as the Standard Deviation (STD) of the range domain to perform image denoising. These parameters are commonly known as the denoising strength. The greater the denoising strength, the stronger the denoising effect is. Extreme denoising strength will cause the spatial domain to be suppressed. An adequate guess of the standard deviation of the image noise is needed to perform denoising to prevent spatial domain information from being removed. Recent developments in image denoising involve machine learning approaches such as U-net, but the downside for such an approach is the data-driven image recovery and the amount of data required. Classical denoising algorithms such as Block Matching 3D (BM3D) and total variation denoising require prior information input as the parameter for denoising [12,13]. Noise strength estimation is important as we can use this method to perform a correlation study for the type of noise, the strength of the noise, and the suitable parameter to be used for the image denoising algorithm.

In this paper, we propose Distortion Estimation using the image Gradient (DE-G). We performed feature extraction from the noisy image by comparing the feature space with the ground truth–noisy image pair. In this research, we show that DE-G can estimate the noise strength of any given noise model accurately. We categorized noise as additive and multiplicative noise and conducted noise parameter estimation using DE-G to estimate the parameters of the noise model. Next, we further extended our work to estimating multiple distortions and showed the capability of our proposed method to estimate combined distortion.

The remainder of this paper is organized as follows. Section 2 gives the literature review. Section 3 explains the need for noise parameter estimation and the proposed method (DE-G) in detail. Section 4 presents the results and discussions. Section 5 gives the conclusion and the usage of DE-G.

## 2. Literature Review

Noise is a major source of corruption in images. A noisy image can cause information loss. Noises can be classified into two major classes: additive noise and multiplicative noise. Additive noises are noises that are added to the image such as Gaussian noise. Multiplicative noise is multiplied with the ground truth image such as speckle noise [14]. Other distortions such as blurring are modeled by taking the convolution of the point spread function with the ground truth image [15]. The point spread function is a 2-dimensional array describing the radiation intensity distribution in the image of a point source [15].

We name these corruptions as noise and distortion respectively in this paper. Reference [14] discussed noise models such as Gaussian noise, salt-and-pepper noise, and grain noise. Reference [16] updated some noise models that have been added throughout the years and provided a quick review of the noise models. Reference [17] proposed a Monte Carlo simulation approach for rendering these film grain noises using a Poisson distribution and other algorithms.

X-rays are commonly used in medical images such as Magnetic Resonance Imaging (MRI) and Advance X-ray Imaging (AXI) for silicon defect detection. X-ray doses are desired to be as low as possible to reduce harm towards the subject, but as the dose decreases, the X-ray noise increases. These noises are known as quantum noise and are often modeled with Poisson noise [18]. Electronics’ noises were described by [19] as false bit-flips due to thermal heating in electronics. Salt-and-pepper noise is used to model these false bit-flips [20]. Reference [20] also presented a way to perform a median filter at the circuit level to remove such noise in an image.

### 2.1. Noise Parameter Estimation

Noise can be mathematically modeled, and its strength is dependent on the input parameters, for example random noise that is modeled using a Gaussian distribution. As the standard deviation of the Gaussian noise model increases, the image will suffer from a noisier corruption. Noise parameter estimation estimates these noises accurately at a high repeatability. Noise parameter estimation is important for the correct noise modeling of the image such that the latent noisy image is not overly noisy and close to reality.

Reference [21] proposed a noise estimation method by using Bayesian MAP inferencing on a distorted image and performed a review on noise modeling in a CCD camera. The authors proposed that noises that are seen from the images are mostly from the propagation stage. Noise can be irradiance-dependent or independent noise. These noises are additive and passed through a Camera Response Function (CRF). The authors ignored the additive noise that is added to the image after the image passes through the CRF as they claimed that with modern-day cameras, additive noise that affects the image is very small. One of the most widely used methods for estimating noise is through the Mean Absolute Deviation (MAD). However, this method is commonly used in local and smooth kernels. Reference [22] decomposed an image into the wavelet components and trained with a set of 13 images. Reference [22] used wavelet transform as a feature extractor from the degraded image and used these features to fit a final output value. However, the model can only predict noise parameters that are in the training dataset and is not as versatile asother noise models.

The median absolute deviation counters the idea of the mean absolute deviation in estimating noise [23]. The median absolute deviation is more robust than the mean absolute deviation and is less affected by outliers, which makes the edges in non-smooth regions less affected by the overall score. Reference [24] challenged the median absolute deviation method and proposed the Residual Autocorrelation Power (RAP). The RAP can estimate the additive noise standard deviation to a high level of accuracy when compared with the median absolute deviation. Reference [25] suggested that the noise estimation of colored images and grayscale images is different as colored images have multiple channels to take under consideration, while grayscale images, which are commonly used in the medical field, have a single channel. Reference [26] used a multivariate Gaussian noise model to visualize the pixel spread of each channel and estimate the covariance of noise by comparing each channel. Through an iterative process, the noise covariance was estimated. Reference [27] used the mean deviation at a certain Region Of Interest (ROI) and computed the estimated standard deviation of the Gaussian noise added to the image. The result showed high consistency and accuracy at low-level noise (σ = 5), but started to deviate at higher noise levels (σ = 10, σ = 15). Reference [28] performed a blind quality assessment using the method of moments to measure the signal-to-noise ratio of the one-dimensional signal of a cosine waveform. Reference [29] used a statistics-based approach for noise estimation using the skewness of the image pair and estimated the strength of the noise of the Additive White Gaussian Noise (AWGN). Reference [30] performed noise estimation on mammograms by using Rician noise modeling and used the estimated value to perform image denoising using a nonlocal mean denoising filter.

Noise study and strength estimation have been given much attention because noise allows researchers to have a better understanding of the environment that causes noise and the effect of fine-tuning these physical parameters’ relationship on the image quality. Much work has been performed in noise estimation working on specific noises such as X-ray noise, described by different distributions, and general noise, modeled using Gaussian noise. The ability of these methods to predict other forms of noise has not been discussed formally, and their capability of estimating combined noise has also not been explored.

### 2.2. Usage of Parameter Estimation

An accurate prediction of the noise level from the image allows for less human intervention in performing image processing and environment physics study. Classical image denoising algorithms are widely used in performing image recovery. Image denoising requires some prior information from the user to perform image denoising. The Gaussian low-pass filter is one of the most well-known filters that removes high-frequency noise from the image, but the structural integrity of the image will be sacrificed when the denoising strength is too strong. An improved version of the Gaussian filter is the bilateral filter, which takes a spatial standard deviation and a range standard deviation. The bilateral filter is a nonlinear and edge-preserving denoising algorithm, but the denoising strength must be set correctly or it will approximate the Gaussian blur [31]. The adaptive Wiener filter and the Modified Median Wiener Filter (MMWF) perform denoising by taking the noise variance of the image [32,33]. BM3D requires the user to input a standard deviation that directly relates to the noise level of the image [12]. These denoising algorithms are very popular in the medical imaging chain. The input parameters of the denoising algorithm have a high correlation with the noise model parameters. Another image processing algorithm that needs the noise information is image segmentation. Reference [34] used a fuzzy C-means clustering algorithm to perform image segmentation on images that were corrupted by impulsive noise. The method can be further improved by providing the algorithm with the noise information. Hence, noise information is crucial in performing image processing algorithms.

The root cause of noise can be due to the physics of the surrounding environment or during the conversion of photons to pixel values in the camera. Conventionally, brighter pixels are due to more photons received by the camera sensor during the shot duration. The camera setting can significantly affect the image quality, for example increasing the shutter speed will reduce the exposure time. Noise will become more apparent compared to the overall signal received. The overall Signal-to-Noise Ratio (SNR) [35] will decrease with increasing exposure time. Light travels differently at different frequencies. High-frequency light will be able to penetrate opaque objects and allow us to see through the object. A well-known example of the real-life usage of these properties is in defect detection using X-rays in AXI. The X-ray projection noise is caused by the scattering of X-ray photons when they pass through an object. The scattering angle is dependent on the energy of the X-rays (frequency) and the type of material being used as the X-ray source [36]. Hence, the study of the noise model parameter can be of great help in determining the noise level of the X-ray projection image and selecting an optimal standard deviation for the denoising algorithm without the need for human intervention.

### 2.3. Image Quality Assessment

The image gradient gives more insight into the physical structure of an image by showing the edges of an image using filters such as the Sobel, Prewitt, and Laplace filter. This gives rise to the idea that the image gradient can be used to measure the image quality. For example, blurred images have less edge information than clear images. Reference [37] proposed image quality assessment using gradient similarity, which utilizes the image gradient to compute an overall score from the luminance, contrast, and structure. Reference [38] proposed the Gradient Magnitude Standard Deviation (GMSD), which calculates the standard deviation from the quality map of a gradient of the image. Structural Similarity Index Measure (SSIM) compares the ground truth image with the restored image with three metrics, luminance, contrast, and structural similarity; all three metrics are consolidated into one overall score [39]. The Multi-scale SSIM (MS-SSIM) extends the idea of the SSIM and changes the final consolidation score formula to obtain a more accurate grading [40]. Information-Weighted SSIM (IW-SSIM) also extends the SSIM by adding a weighted pooling to the SSIM [41]. These image quality assessments show a high correlation with the mean opinion score of humans. However, the type and strength of distortion of the image is not specifically estimated.

The Laplacian of Gaussian (LoG) and the Difference of Gaussian (DoG) pass the images through a Gaussian blur, effectively removing noise in the image and comparing the structural difference between the reference image and the filtered image [42]. Recent advancements include using visual saliency, chrominance, and gradient magnitude to grade an image in CIELAB, as proposed by [43]. Reference [44] proposed the SuperPixel-based SIMilarity Index (SPSIM), which obtains a set of image pixels that have the same visual characteristics, and called them superpixels. Each superpixel is graded with the superpixel luminance similarity, superpixel chrominance, and gradient similarity.

The Human Visual System (HVS) is extremely complex at perceiving information from the real world and transmitting the scenario that we see in our daily lives to our brain for processing. Image quality assessment grading is performed by comparing the objective scores of the subjects (participants in the experiment) and the given score of the image quality assessment. The free-energy principle would suggest that when a human sees a corrupted image, he/she will have a mental picture of how clean the image should look [45]. By comparing the mental picture and the given corrupted image, humans can give a score to the corrupted image. The Mean Opinion Score (MOS) and Difference Mean Opinion Score (DMOS) are two common metrics computed with the result obtained from the human subjective experiment [46]. To mimic the complex HVS, visual saliency is used to put more emphasis on the foreground of the image, rather than the background of the image. Reference [47] proposed using deep neural networks for both no-reference and full-reference image quality assessment. Before the pooling layer arrives at the final score, a fully connected neural network is used to estimate the weights of each patch. Reference [48] proposed using an attention residual network for full-reference image quality prediction, which maps out the saliency for final grading.

We classify these image quality assessments into two different types of distortion: (i) noise and (ii) structural difference. Noise includes all types of noise, Gaussian noise, Poisson noise, etc, and structural distortion includes Gaussian blur, compression distortion, etc. Image quality assessment is considered as an overall score for the noisy image, and the score is obtained through the combination of the information retained, feature similarity, etc. Image quality assessment can be used as a guide for designing a good feature extractor. Image quality assessment that is based on the image gradient exhibits a good response towards noise such as in the GMSD. The noisier the noisy image is, the greater the GMSD score. Hence, we used the image gradient as a method of feature extraction for estimating the noise parameters.

## 3. Methodology

**Figure 1:**
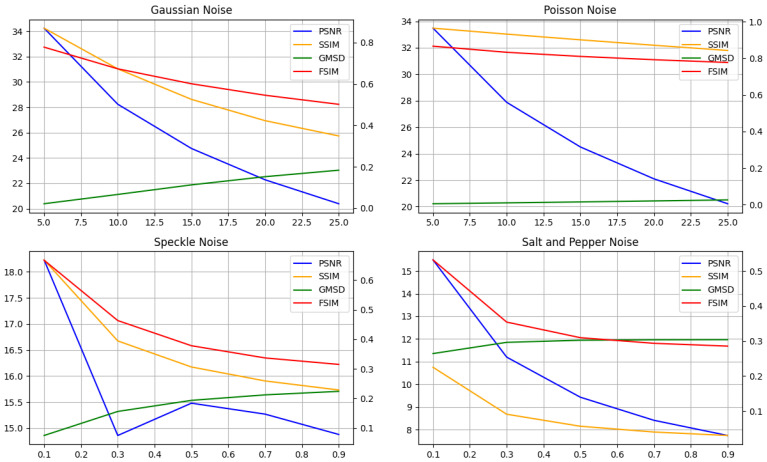
Comparison of IQA with respect to the noise parameter.

We compared the commonly used Image Quality Assessment (IQA) with the predicted value to observe the linearity and performance of IQA in estimating the image score for different noise types. Table 1 shows the simulated result of different IQAs with different noise models. The experiment was conducted using the Kodak image dataset. We compared the linearity using the Peak-Signal-to-Noise Ratio (PSNR), Structural Similarity (SSIM), Gradient Magnitude Standard Deviation (GMSD), and Feature Similarity (FSIM). The numerical value of the IQA score reflects the severity of the corruption of the noise model. Gaussian noise shows a good linearity performance between the IQA scores and the strength of the noise. However, the other noise model does not have the same linearity performance as Gaussian noise. In Poisson noise, the PSNR still shows a good description of the noise strength, but other IQA scores are not as sensitive as the PSNR. Speckle noise and salt-and-pepper noise have a much lower PSNR value compared to Gaussian and Poisson noise, and the PSNR curve is damped faster. The GMSD of both speckle and salt-and-pepper noise saturate very fast compared to Gaussian and Poisson noise. The plot of Table 1 is shown in Figure 1. This shows that IQA gives a good overall score of the image and is able to aid in predicting Gaussian noise. However, the performance of IQA in noise prediction is not recommended.

### 3.1. Noise Estimation Modeling

All image quality assessment methods of scoring are the consolidation of different types of distortions applied to an image. We aimed to create an algorithm that extracts the distortion information effectively from the noisy image statistically. DE-G provides the information of the dominant and secondary distortions in the resulting image. The proposed method is not restricted to the size of the image and can account for any known distortion that can be described through mathematical modeling. Besides, the proposed method can also account for multiple combined distortions.

Reference [49] showed that natural images exhibit a generalized Gaussian curve when taking the pixel count of the derivative of the image. Reference [49] used this property to perform image deblurring by curve fitting using the K number of the Gaussian distribution, while [50] used a piecewise function to model the image gradient property of natural images. This property inspired us to observe image information in a statistical viewpoint, and we observed that different noises will result in different image gradient responses. Instead of sacrificing the accuracy, we adopted a full-reference approach, which requires the input of a ground truth image to be used as a reference.

Distortion models usually have one or more parameters to set the distortion properties. Gaussian noise consists of two parameters, the mean and the standard deviation. The mean sets the luminance of the noise, while the standard deviation describes the probability of the pixel spread. A higher standard deviation in Gaussian noise results in a noisier image. The mean of Gaussian noise is set to zero as the luminance is normally unchanged when the image is corrupted by noise. Image blurring is modeled with Gaussian blur, and it takes one parameter, which is the kernel size of the blur function. The larger the kernel size, the more severe the blurring effect is. Noisy images are described as shown in Equation (1), where the image we received is *f* and the noise is *n*.

(1) f=g+n

Another type of distortion that might occur during the imaging chain process is at the CRF where the camera is out of focus or experiences motion blur. These distortions have a convolutional relationship between the ground truth image and the CRF. Assuming that noise is temporal, *n* is additive after the convolution. Hence, we modeled the equation for the received image as shown in Equation (2):

(2) f=h∗g+n

where *h* is the CRF and *n* is the additive noise. Note that noise can be additive or multiplicative, but here, we describe the mathematical modeling of a distorted image in an additive manner. We then took the derivative of the image and plotted the pixel spread from −255 to 255. The details of the image gradient formulation are shown in Appendix A.

The derivative of an image is obtained by using Equations (3) and (4):

(3) $$I_{x}\left(i,j\right)=I\left(i+1,j\right)-I\left(i,j\right)$$

(4) $$I_{y}\left(i,j\right)=I\left(i,j+1\right)-I\left(i,j\right)$$

where i,j∈W+1,H+1. *W* and *H* are the width and height of the image. $I_{x}$ and $I_{y}$ are zero padded at the left end and bottom end of the image, respectively. $I_{x}$ is the resultant partial derivative map across the width, and $I_{y}$ is the resultant partial derivative map across the height. Note that the partial derivative is computed separately and shown separately as there are cases where we can detect noise on the X-axis or the Y-axis.

### 3.2. Image Gradient Response

**Figure 2:**
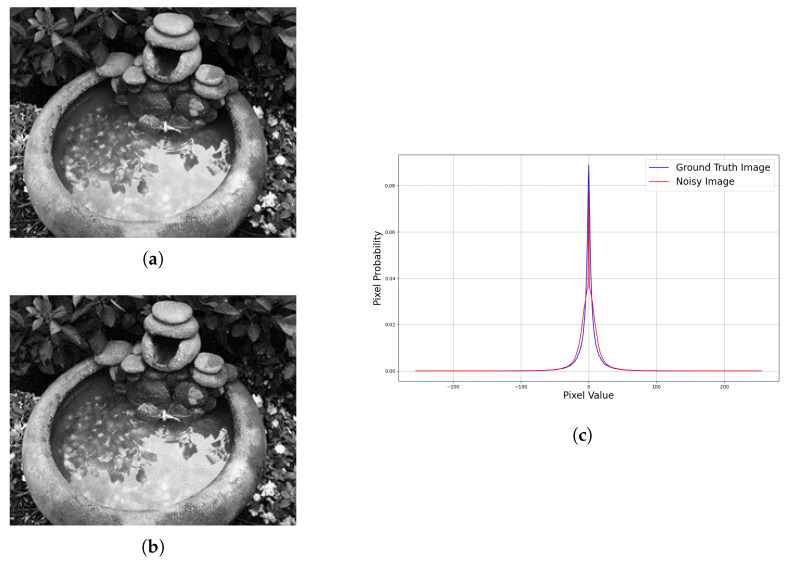
Ground truth–noisy image pair with their respective image gradient statistics. The image gradient statistics are very close to each other, which are similar to the original image, but the features are clearly described through the image gradient statistics. (**a**) Ground truth image. (**b**) Noisy image. (**c**) Image gradient statistics along the X-axis.

Noise strength is hard to estimate through human judgment. Figure 2a is the ground truth image and Figure 2b is the noisy image with Gaussian noise of zero mean and standard deviation of five, respectively. The image gradient statistics response in the x direction is shown in Figure 2c. The blue curve represents the ground truth image, while the red curve represents the distorted image.

**Figure 3:**
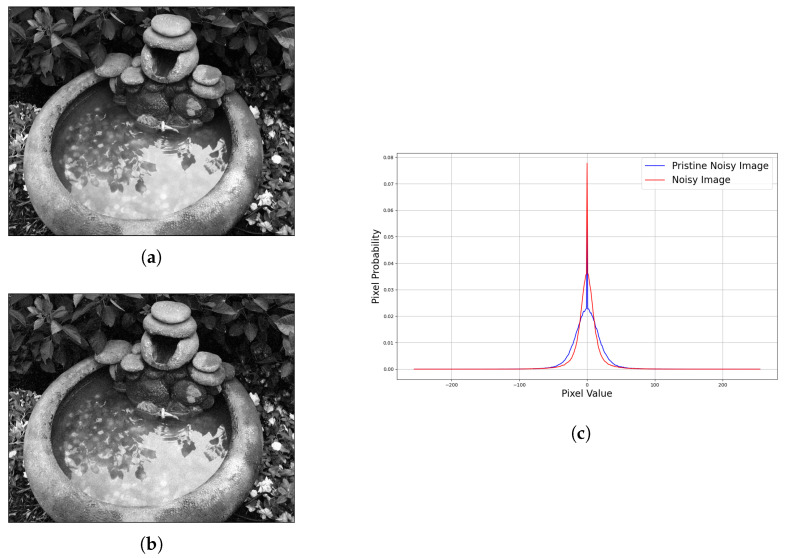
Comparison of image gradient statistics of the pristine noisy image and latent noisy image. The pristine noisy image has a standard deviation of 10, while the latent noisy image has a standard deviation of 5. (**a**) Latent distorted image, standard deviation of 5. (**b**) Pristine distorted image, standard deviation of 10. (**c**) Image gradient statistics along the X-axis.

Then, we conducted a study to estimate the standard deviation of Gaussian noise using the image gradient. A ground truth image was first injected with Gaussian noise with a known standard deviation. We call this a pristine noisy image. We then injected noise into the same ground truth image with a guessed standard deviation. We call this the latent noisy image. The latent noisy image as shown in Figure 3a has a standard deviation of 5, while the pristine noisy image has a standard deviation of 10 (Figure 3b). The image gradient statistics is shown in Figure 3c. The comparison of the pristine noisy image of standard deviation 10 and the latent noise image of standard deviation 5 shows that the greater the standard deviation of the noise model is, the greater the suppression in 0. By increasing the guessed standard deviation closer to 10, the two curves will become closer to each other. When the curve fit each other, the standard deviation of the latent noisy image is the same as the pristine noisy image.

To evaluate the image gradient response of the algorithm, we used an open-source dataset to validate our claim. The LIVE dataset consists of ground truth–noisy image pairs where the noisy images were created using 5 different distortions [39,46,46]:

1. JPEG compressed images;
2. JPEG2000 compressed images;
3. Gaussian blur;
4. White noise;
5. Fast-fading Rayleigh channel.

**Figure 4:**
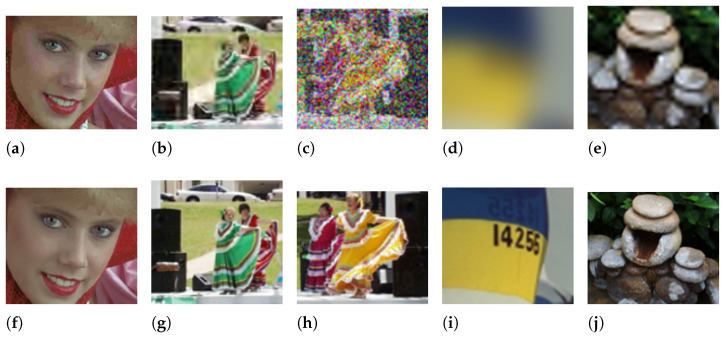
Corruption from the LIVE dataset. The corruption is performed on the entire image. The cropped noisy and ground truth pair image is shown in (**a**–**j**). The noisy image is in (**a**,**b**), while (**f**–**j**) is the respective ground truth image. The noisy images consist of (**a**) JPEG2000 compression, (**b**) JPEG compression, (**c**) white noise, (**d**) Gaussian blur, and (**e**) the fast-fading Rayleigh channel.

For each of the distortions, we observed a different image gradient response. Figure 4a–e shows the example of the degradation for each image corruption from the LIVE dataset. The ground truth image of each responding noisy image is from Figure 4f–j. Gaussian blur and the fast-fading Rayleigh channel showed a very similar distortion as they significantly reduced the image frequency. This is the reason why this caused the gradient statistics to tighten at a higher frequency. JPEG compressed images have blurring at some regions, but also, the frequency of the image increases around the blurred region. This is observed from the vegetation in the background of the dancer and added artifacts on the stairs. JPEG2000-compressed images pixels are highly pixelated from the compression, and this will cause the image gradient to be reduced. However, the overall image is still maintained, and this causes the image to have a tight neck at a relatively lower frequency, but not at a higher frequency. This is in contrast to Gaussian blur and the fast-fading Rayleigh channel, which remove the high-frequency elements from the image. White noise consists of random noise having the properties of a Gaussian distribution. The image is corrupted with severe white noise, which causes the gradient distribution to be flattened. The center still maintains the sharpest peak, as there is still some information remaining in the image.

**Figure 5:**
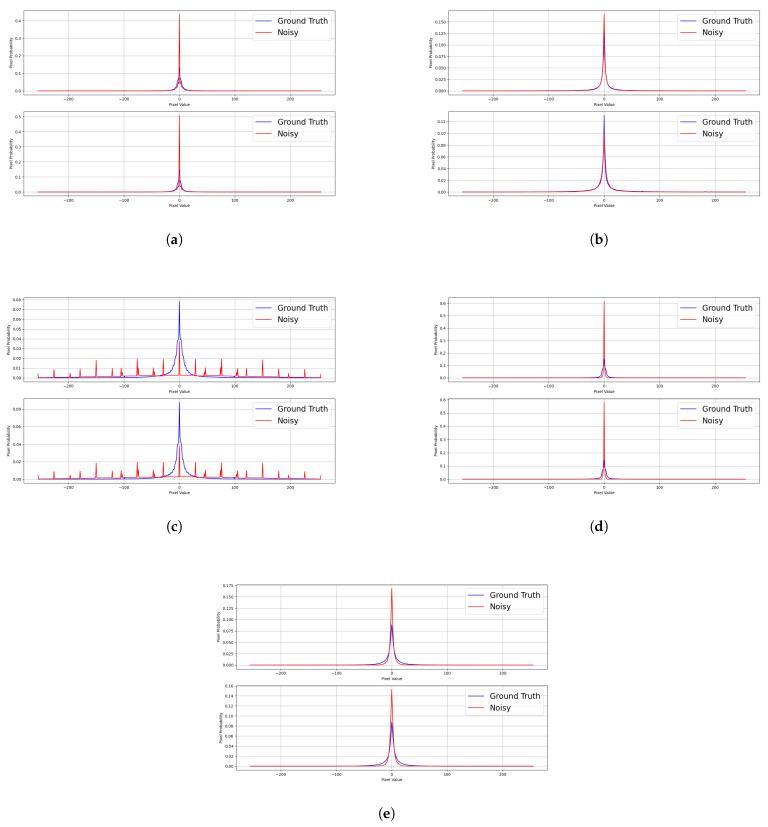
Image gradient distribution for each type of distortion in the LIVE dataset. The upper graph is the image gradient in x, while the lower graph is the image gradient in y. Cases: (**a**) JPEG2000 compression, (**b**) JPEG compression, (**c**) white noise, (**d**) Gaussian blur, and (**e**) fast-fading Rayleigh channel.

From Figure 5a–e, the noise-corrupted images are JPEG2000-compressed images, JPEG-compressed images, Gaussian blur, white noise, and the fast-fading Rayleigh channel. The upper graph shows the image gradient statistics of the horizontal gradient, while the bottom graph shows the image gradient statistics of the vertical gradient. The blur line is the ground truth image, and the red line is a noisy image. JPEG2000-compressed images tighten the lower neck of the distribution, but the rest of the distribution remains relatively unchanged. JPEG-compressed images have the response of widening the neck of the distribution, but lower near 0. White noise consists of random spikes in the noisy image distribution, and we noticed that the distribution is not only symmetric, but the gradient distribution in X is similar in Y, despite the difference in the X and Y distribution of the original image. Gaussian blur is very similar to JPEG compression and is tightened in the lower upper neck, but the gradient near 0 remains relatively unchanged. Only the large gradient difference is affected. The fast-fading Rayleigh channel shows a significant shift from the high gradient difference to the low gradient difference. The neck of the distribution is significantly tightened, and the tip is lifted.

In order to quantify the amount of loss between two curves, a cost function is needed to measure the Area Under the Curve (AUC) of the blue (ground truth) and red (noisy) curve. The greater the area, the greater the loss of the latent noisy image is. The loss function we chose to use was the Canberra distance. The Canberra distance is the measurement of the distance between the two curves. The formula for the Canberra distance is shown in Equation (5). The Canberra distance was chosen because it provides a bounded value from 0 to 1 for each extracted feature.

(5) $$d=\sum \frac{|x_{n}-x_{t}|}{|x_{n}\left|+\right|x_{t}|}$$

where *d* is the Canberra distance, $x_{n}$ is the noisy image, and $x_{t}$ is the ground truth image. The overall algorithm for DE-G is as shown in Algorithm 1. The first step is taking in a pristine noisy image, *T*, and a latent noisy image, *X*. Then, the size of the image is determined. Both images are passed to the feature extractor through the image gradient and return four image gradient distributions:

1. Image Gradient with respect to *x* of the pristine image, $\delta_{x}\left(T\right)$;
2. Image Gradient with respect to *y* of the pristine image, $\delta_{y}\left(T\right)$;
3. Image Gradient with respect to *x* of the latent image, $\delta_{x}\left(X\right)$;
4. Image Gradient with respect to *y* of the latent image, $\delta_{y}\left(X\right)$.

### 3.3. Noise Type Response Using the Proposed Feature Vectors

**Figure 6:**
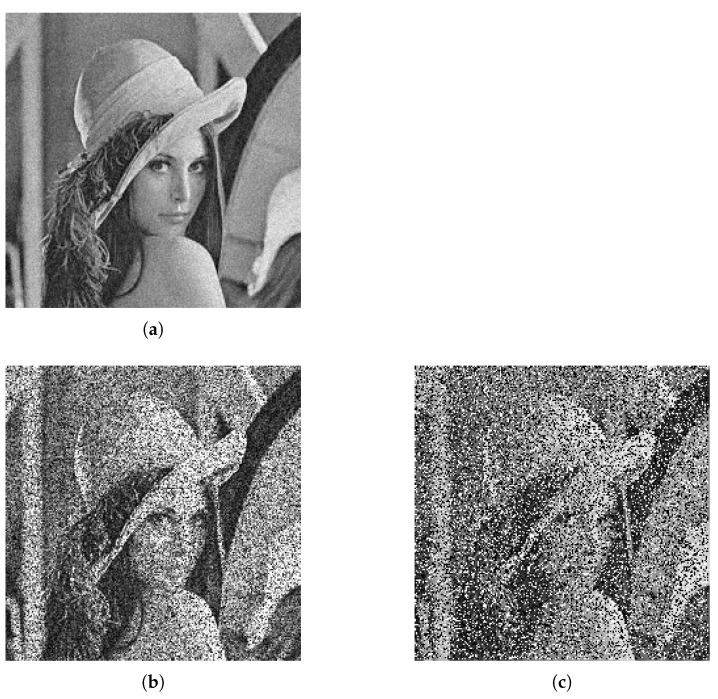
Noisy image corrupted with Gaussian noise (**a**), speckle noise (**b**), and salt-and-pepper noise (**c**). It is hard to differentiate speckle noise from salt-and-pepper noise by the human eye.

**Figure 7:**
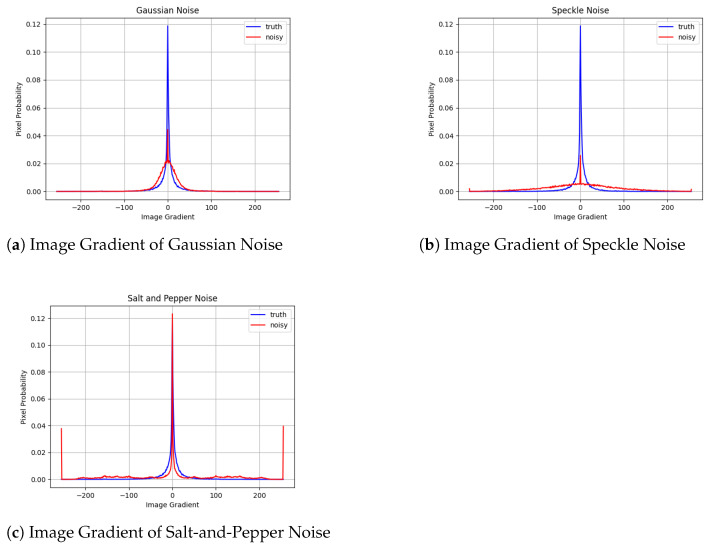
Image gradient response. (**a**) is the image gradient of Figure 6a (red) compared to the clean image (blue). (**b**) is the image gradient of Figure 6b (red) compared to the clean image (blue). (**c**) is the image gradient of Figure 6c (red) compared to the clean image (blue). Note that the extracted features clearly differentiate each noise type.

Since the image gradient pixels range from −255 to 255, the total feature vectors at the X and Y axis is 1022. Using a clean image (Lena), we augmented the clean image with several noise models. The noise models included additive noise (Gaussian noise and Poisson noise), multiplicative noise (speckle noise), and impulse noise (salt-and-pepper noise). Gaussian noise was set to a fixed mean value and a variable standard deviation. Poisson noise took exactly one input to manipulate the noise spread, which was the variance. We altered the speckle noise by adding a weighting coefficient to the speckle noise, as shown in Equation (6):

(6) f=g+g×N(0,1)×w

where *w* is the weightage that controls the speckle noise strength. The salt-and-pepper noise has two parameters, which are the ratio of salt-to-pepper and the number of events of false bit-flips. Using the proposed feature extractor, we observed the image gradient response of the three types of noise models discussed above. The clean image was corrupted with Gaussian noise (Figure 6a), speckle noise (Figure 6b), and salt-and-pepper noise (Figure 6c). Figure 7a is the image gradient response of Figure 6a. Figure 7b is the image gradient response of Figure 6b. Figure 7c is the image gradient response of Figure 6c.

### 3.4. Noise Strength Prediction

To estimate the noise strength, the Canberra distance was used as a loss function by taking the sum of the distance between $\delta_{x}\left(T\right)$, $\delta_{x}\left(X\right)$ and $\delta_{y}\left(T\right)$, $\delta_{y}\left(X\right)$ to obtain $\Delta_{x}$ and $\Delta_{y}$, respectively. The $L_{2}$ of $\Delta_{x}$ and $\Delta_{y}$ was taken as the final loss function. For colored image, the $L_{p}$ of $\Delta_{x}$ and $\Delta_{y}$ was taken, where *p* depends on the subpixel channel. The overall algorithm for DE-G is shown in Algorithm 1. Our proposed method requires several pieces of a priori knowledge such as the ground truth image, the noisy image, and the noise model. The optimum noise parameters are estimated through each iteration.
**Algorithm 1** Algorithm for the loss function to estimate the noise level.INPUT(X,T)  W,H←shape(X)  $\delta_{x}\left(T\right)\leftarrow T\left(i+1,j\right)-T\left(i,j\right)$ **for** i,j∈W,H  $\delta_{y}\left(T\right)\leftarrow T\left(i,j+1\right)-T\left(i,j\right)$ **for** i,j∈W,H  $\delta_{x}\left(X\right)\leftarrow X\left(i+1,j\right)-X\left(i,j\right)$ **for** i,j∈W,H  $\delta_{y}\left(X\right)\leftarrow X\left(i,j+1\right)-X\left(i,j\right)$ **for** i,j∈W,H  $\Delta_{x}\leftarrow \sum \frac{|\delta_{x}\left(X_{i}\right)-\delta_{x}\left(T_{i}\right)|}{|\delta_{x}\left(X_{i}\right)\left|+\right|\delta_{x}\left(T_{i}\right)|}$ **for** i=−255,⋯,255  $\Delta_{y}\leftarrow \sum \frac{|\delta_{y}\left(X_{i}\right)-\delta_{y}\left(T_{i}\right)|}{|\delta_{y}\left(X_{i}\right)\left|+\right|\delta_{y}\left(T_{i}\right)|}$ **for** i=−255,⋯,255  $loss\leftarrow L_{2}\left(\Delta_{x},\Delta_{y}\right)$  **return** loss

**Figure 8:**
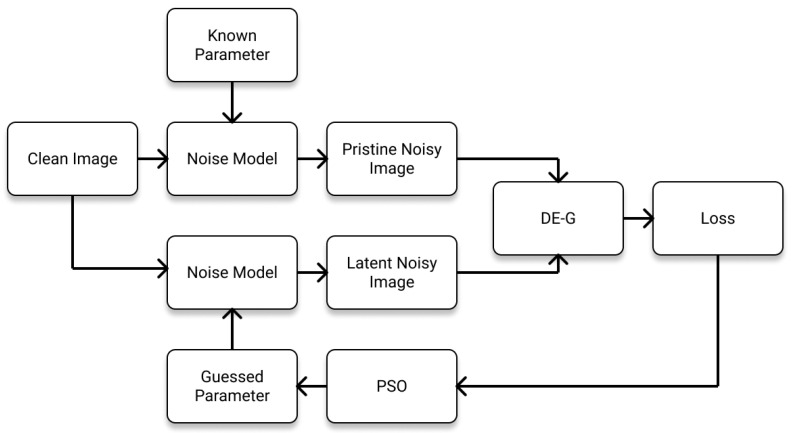
Block diagram for noise parameter estimation. The clean image is the ground truth image. To demonstrate the capability of DE-G in estimating accurate noise parameters, the clean image is injected with noise with a known set of parameters to produce the pristine noise image. The clean image is then injected with noise with a set of guessed parameters. The pristine noisy image and the latent noisy image are compared using DE-G. Through each iteration, the guessed parameters are updated using Particle Swarm Optimization (PSO). The algorithm ends after the maximum iteration number of PSO is reached.

We used the Kodak image dataset to perform the evaluation of the estimated noise parameters. We classified the conducted test into four types of distortion estimations: additive noise estimation, multiplicative noise estimation, impulsive noise estimation, and combined noise estimation. The Kodak images consist of natural images that have a size of 768 × 512 or 512 × 768. We resized the images to 192 × 128 or 128 × 192 depending on the original image size. We used Particle Swarm Optimization (PSO) to estimate the noise parameters. To prevent heavy oscillation, we adopted a batch mean strategy by taking the mean loss of 20 noisy image batches. The overall algorithm is shown in Figure 8. In real-life usage, the pristine noisy image is the noisy image in the ground truth–noisy image pair instead of noise injection into the clean image.

## 4. Results and Discussions

The experiment was conducted by injecting noise with a known parameter and attempting to estimate the injected noise parameter. The parameter of the intended noisy image is referred to as the ground truth parameter, and the noisy image is known as the pristine noisy image. The predicted parameter is known as the estimated parameter, and the predicted noisy image is known as the latent noisy image. The mean and the standard deviation of the ground truth parameter and the predicted parameter were calculated. We also evaluated the Mean-Squared Error (MSE), Mean Absolute Deviation (MAD), and the error percentage between the predicted value from DE-G and the known ground truth (Mean Relative Error Rate (MRER)). We also compared the accuracy of the Canberra distance with the KL divergence for all four scenarios. The KL divergence is a commonly used loss function to quantify the difference between two probability distributions [51].

### 4.1. Parameter Estimation of Additive Noise

Additive noise describes that noise that has an additive nature in the image. The additive nature of noise means that noise is independent of the image pixel value. Gaussian noise is the most-often found noise in images. Random noise in images might be caused by the surrounding radiation, random processes in the camera, or random error in the image. The mean of Gaussian noise is commonly set to zero because noise does not have a DC component to offset the pixel value of noise. We conducted the estimation of Gaussian noise with a mean of 0 and a standard deviation of 5, 10, 15, and 20. The results are shown in Table 2. Our proposed method was compared with [29] in predicting the standard deviation of Gaussian noise. The method of [29] does not show the capability of predicting other noise models. In the next subsection, we show the ability of DE-G in predicting other noise models such as multiplicative noise, impulsive noise, and combined noise.

### 4.2. Parameter Estimation of Multiplicative Noise

An example of multiplicative noise is speckle noise. Speckle noise strength is dependent on the pixel value. Speckle noise follows the normal distribution:

(7) $$X∼N(\mu ,\sigma^{2})$$

Comparing Equations (6) and (7), we obtain μ=g and $\sigma^{2}=w\times g$. The mean and the variance of the normal distribution are related to the original ground truth image. Speckle noise is commonly found in radar and medical ultrasound images. It is caused by the coherent processing of back-scattered signals from multiple distributed targets. DE-G can predict the weighting factor, *w*, of speckle noise, and the results are shown in Table 3.

### 4.3. Parameter Estimation of Impulsive Noise

Salt-and-pepper noise is noise caused by thermal noise in electronics. Energy is lost in electronic components as heat energy. As heat energy is not efficiently ventilated, the heat energy will cause a high temperature in the circuit. The high temperature of the circuit will cause the bit value to malfunction and result in false bit-flips. The false bit-flips will result in random pixels in the image to be white (salt) or black (pepper). Salt-and-pepper noise will significantly change in the tail of the image gradient response. The salt-and-pepper noise is modeled with two parameter inputs, the ratio between salt-and-pepper, ranging from (0, 1), and the ratio of defect pixel to the total amount of pixels in the image. In this study, the ratio of salt-and-pepper was varied from 0.2 to 0.7 and the amount of salt-and-pepper was predicted. The results are shown in Table 4.

### 4.4. Parameter Estimation of Combined Corruption

Realistic images consist of several distortions applied to the image. As described in Equation (2), the received image can consist of a convolutional distortion and noise. We used Gaussian blur and Gaussian noise as our distortion combination. Gaussian blur is commonly found in images when the image is out of focus. The recovery process requires a known window size to perform the inverse process. Gaussian blur is not considered as noise that is added to the clean image, but a convolutional distortion. Gaussian blur had one parameter, which is the blur window size. The larger the window size, the greater the blur strength. We denote the window size as *W* and the standard deviation of the Gaussian noise as σ. The true window size and standard deviation have a subscript of *t*. In Section 4.1, Section 4.2, Section 4.3 and Section 4.4, parameter estimation was performed using the Canberra distance and Kullback–Leibler divergence. The Canberra distance was chosen for the parameter estimation for combined noise because of the stability of the Canberra distance in predicting noise. The results are shown in Table 5. The predicted parameters in Table 5 are the mean of the predicted parameters in the Kodak dataset.

### 4.5. Discussions

In this section, we discuss the results we obtained through our experiment in estimating the noise strength. The Canberra distance was used for DE-G in predicting noise parameters and showed a stable and accurate result. Our method is not restricted by image size, nor the noise type. We were able to predict the Gaussian noise standard deviation with a lower MSE than [29], but with a higher error rate of 6% for the Canberra distance and 4% for the KL divergence. However, we leveraged this issue through the capability of DE-G in predicting other noise models using a unified algorithm. DE-G can predict speckle noise and salt-and-pepper noise with an error rate of less than 3% for the Canberra distance and 5% for the KL divergence. Due to this reason, we chose the Canberra distance as the loss function for the combined corruption parameters’ estimation. We showed the capability of DE-G in estimating the noise parameters for Gaussian blur and Gaussian noise with error rates of 7% and 10%, respectively.

Previous work focused on a specific type of noise, and the approaches were through looking at the power spectrum, frequency domain, and statistical viewpoint. Such methods restrict the algorithm to predict only one type of noise. DE-G is able to predict different noise types and combined corruption, which proves to be useful and highly desirable, as real-life images consist of more than one noise source.

As shown in Section 4.1, Section 4.2, Section 4.3 and Section 4.4, we showed the capability of DE-G in estimating noise parameters of different noise models. Our method is not restricted to any noise model and is able to predict multiple parameters as the output for any known noise model. Instead of having a correlated SNR or aggregated score such as in IQA, we would argue that our method is very useful for researchers to study the nature of noise. This is because with the prior knowledge of the noise model, our method can neglect the other distortions applied to the image. This is important because real-life imaging systems consist of more than one type of noise present.

The proposed method comes with several restrictions, the requirement of the the ground truth–noisy image pair and the prior knowledge of the noise type. Some ground truth–noisy image pair are hard to obtain due to the limitations of the process of data collection. Data acquisition has become an expensive and tedious task for researchers. Our proposed method will aid in the process of data collection by performing realistic data augmentation with accurate noise model parameters. The process of data acquisition can be minimized after the user has obtained a confident noise model parameter using DE-G. The ground truth image can also be obtained through image processing techniques without losing any information such as image averaging [52]. DE-G can be used to study the expected noise model parameters. Synthetic noisy images that mimic real–life images can be obtained by injecting noise into the averaged image. Next, noise models are often well-established, and our proposed method can also be used in researching new noise models by proving the loss.

## 5. Conclusions

Images are becoming one of the useful forms of information transfer between the real world and the digital world. However, the corruption by noise is one of the major threats that reduces image quality. The proposed method, DE-G, effectively extracts noise information from the image pair by employing the Canberra distance as the loss function. DE-G is able to predict Gaussian noise (additive) with an MRER of 6%, speckle noise (multiplicative) with an MRER of 1%, and salt-and-pepper noise (impulsive) with an MRER of 3%. DE-G is also able to perform combined corruption (Gaussian blur and Gaussian noise) parameters’ estimation with an MRER of 7% and 10%, respectively.

Our work proposed a multi-noise parameter estimation for different noise types, and it is also capable of estimating combined corruption. We proposed a feature extractor that effectively extracts noise information from the ground truth–noisy image pair. This will be useful because our method accounts for any image size. We also proposed a loss function that can estimate the optimum noise parameters from the extracted features.

The nature of noise has a strong relationship with the surrounding environment. Noise strength will change with the settings of the surrounding environment. The most effective way to reduce and prevent noise in an image is to understand the nature of the noises and suppress them by changing the parameters of the surroundings. Noise model parameters can be used as a guide for a specific type of noise. Since the strength of synthetic noise is highly dependent on the noise model parameters, an accurate estimation of the noise model is highly desirable to study the relationship between the physical environment and the noise model parameter. Besides the study of the nature of noise, noise modeling is also very important in image augmentation. Contrastive loss has recently gained much attention from the machine learning community. One of the major parts of contrastive learning is data augmentation. However, the way data are augmented is not specified. We argue that noise augmentation will help in the training process, but the correct range of noise model parameters should be defined clearly according to the application and the physical environment. Ground truth–noisy image pairs are hard to obtain due to several restrictions. A good understanding of the nature of noise and the noise strength is needed to create synthetic data for training purposes. As the number of data increase, the training process can be more robust.

The next step in our research is to address the weakness of DE-G, which is the need for a ground truth image by balancing the accuracy and applicability. References [49,50] showed that natural images exhibit a generalized Gaussian distribution trend and used that property to perform image deblurring. Using a similar approach, we can design a no-reference noise parameters’ estimation algorithm. In Section 3.3, we presented the features’ behavior under different types of noise. Noise-type prediction can also be performed through a machine learning approach. Classical deep learning requires a fixed input to the model, and our feature extraction technique can fix any image size to a fixed size input feature. Next, we will also look into the possibility of using DE-G in performing image quality assessment. We will also aim to improve the parameter estimation algorithm by using a more robust approach such as [53]. Further development of DE-G in the study of the environment, data augmentation, and data generation will be carried out to aid in better algorithm design and the ever-growing machine learning data acquisition. 

## Figures and Tables

**Table 1:** Image quality assessment of different noise types.

Noise Type	Truth	PSNR	SSIM	GMSD	FSIM
Gaussian	5	34.24	0.8699	0.0213	0.7781
	10	28.24	0.6732	0.0662	0.6734
	15	24.75	0.5258	0.1124	0.6011
	20	22.28	0.4228	0.1520	0.5457
	25	20.39	0.3489	0.1834	0.5018
Poisson	5	33.48	0.9644	0.0047	0.8659
	10	27.88	0.9318	0.0095	0.8325
	15	24.51	0.9007	0.0146	0.8100
	20	22.10	0.8710	0.0200	0.7919
	25	20.22	0.8424	0.0257	0.7768
Speckle	0.1	18.23	0.6682	0.0744	0.6672
	0.3	14.86	0.3943	0.1557	0.4631
	0.5	15.48	0.3061	0.1932	0.3780
	0.7	15.27	0.2589	0.2118	0.3367
	0.9	14.88	0.2283	0.2237	0.3148
Salt-and-Pepper	0.1	15.49	0.2250	0.2642	0.5319
	0.3	11.20	0.0908	0.2963	0.3542
	0.5	9.43	0.0564	0.3023	0.3096
	0.7	8.41	0.0399	0.3035	0.2935
	0.9	7.74	0.0300	0.3038	0.2855

**Table 2:** Gaussian noise standard deviation estimation. The mean is set to zero, and the strength of the Gaussian noise is determined by the standard deviation.

$\sigma_{t}$	Heng et al. [29]	DE-G with Canberra Distance	DE-G with KL Divergence
5	4.97	5.03	4.84
10	10.03	9.97	9.65
15	15.09	14.94	14.60
20	20.03	20.12	19.45
MSE	0.0050	0.0049	0.1536
MAD	0.058	0.06	0.3666
MRER	2%	6%	4%

**Table 3:** Speckle noise weight estimation. The strength of distortion is determined by the weight of the speckle noise model as shown in Equation (6).

*w*	DE-G with Canberra Distance	DE-G with KL Divergence
0.3	0.2945	0.2742
0.5	0.5055	0.4737
0.7	0.6961	0.6990
MSE	0.00002	0.00045
MAD	0.0050	0.0177
MRER	1%	5%

**Table 4:** Salt-and-pepper noise parameter estimation.

Ratio	Amount	DE-G with Canberra Distance	DE-G with KL Divergence
0.2	0.3	0.3157	0.2854
0.2	0.5	0.5303	0.4914
0.2	0.7	0.6774	0.7288
0.5	0.3	0.3075	0.2963
0.5	0.5	0.5046	0.4946
0.5	0.7	0.6925	0.7034
0.7	0.3	0.3048	0.2955
0.7	0.5	0.5068	0.4997
0.7	0.7	0.6879	0.6970
MSE		0.0002	0.0001
MAE		0.0120	0.0080
MRER		3%	3%

**Table 5:** Combined noise parameters’ estimation.

True Parameter			DE-G with Canberra Distance
$W_{t}$	$\sigma_{t}$	W	σ
5	5	5.2917	4.9411
5	10	4.7083	9.6002
10	5	11.2500	5.1347
10	10	10.6667	7.5610
MSE		0.5443	1.5325
MAD		0.6250	0.7581
MRER		7%	10%

## Data Availability

Restrictions apply to the availability of these data. Data was obtained from Laboratory for Image & Video Engineering at the University of Texas at Austin and are available from the authors with the permission of University of Texas at Austin.

## Appendix A. Image Gradient Formulation

A corrupted image can be described as shown in (A1):

(A1) f=h∗g+n

where *f* is the corrupted image, *h* is the camera response function, *g* is the ground truth image, and *n* is noise. The image gradient is taken as the first derivative of *f* as follows,

(A2) $$f^{\prime }=g^{\prime }*h+n^{\prime }$$

Next, a difference operator, *D*, is needed to measure the distance between $f^{\prime }$ and $g^{\prime }$ effectively. An example of $D(f^{\prime },g^{\prime })$ is taking the difference between $f^{\prime }$ and $g^{\prime }$. The information from *h* and *n* is not removed, but $g^{\prime }$ is also present in $f^{\prime }-g^{\prime }$, as shown below.

(A3) $$f^{\prime }-g^{\prime }=g^{\prime }*\left(h-1\right)+n^{\prime }$$

To effectively remove $g^{\prime }$ from $D(f^{\prime },g^{\prime })$, we consider $f^{\prime }$ and $g^{\prime }$ as a probability mass function,

(A4) $$P_{1}\left(X=f^{\prime }\right)=\frac{N(f^{\prime })}{W\times H}$$

(A5) $$P_{2}\left(X=g^{\prime }\right)=\frac{N(g^{\prime })}{W\times H}$$

The probability mass function calculates the probability of a given value occurring in the entire image of width *W* and height *H*. This will effectively restrict the feature size because the limit of the image is known. For example, for grayscale images, the image gradient ranges from −255 to 255, which has a feature space of 511. This will hold true given any image size. DE-G uses the Canberra distance as the difference operator to estimate the optimum noise parameter.

(A6) $$D\left(f^{\prime },g^{\prime }\right)=\sum \frac{|P_{1}\left(X=f^{\prime }\right)-P_{2}\left(X=g^{\prime }\right)|}{|P_{1}\left(X=f^{\prime }\right)\left|+\right|P_{2}\left(X=g^{\prime }\right)|}$$

The greater the Canberra distance, the noisier it will be. We extended this idea to estimate the noise strength through minimizing the cost function between the pristine noisy image (the noisy image) and the latent noisy image (the ground truth image injected with the noise of an known parameter). The flowchart of the entire process of noise strength estimation is shown in Figure 8. The program was written with Python 3.7. Note that $D(f^{\prime },g^{\prime })$ has two directions, *x* and *y*. $D_{x}$ is denoted as the image gradient in the *x* direction, while $D_{y}$ is denoted as the image gradient in the *y* direction. According to Algorithm 1, $D_{x}$ is $\Delta_{x}$ and $D_{y}$ is $\Delta_{y}$, respectively. The final loss function is $L_{2}$ for both the *x* and *y* direction. For colored images, $L_{2}$ is taken for each channel.
